# DNA-PK inhibition extends the therapeutic effects of Top2 poisoning to non-proliferating cells, increasing activity at a cost

**DOI:** 10.1038/s41598-023-39649-7

**Published:** 2023-08-01

**Authors:** Taixiang Wang, Alastair H. Kyle, Jennifer H. E. Baker, Nannan A. Liu, Judit P. Banáth, Andrew I. Minchinton

**Affiliations:** Department of Integrative Oncology, BC Cancer Research Institute, 675 W 10th Ave, Vancouver, BC V5Z 1L3 Canada

**Keywords:** Cancer therapeutic resistance, Cancer microenvironment, DNA

## Abstract

Type II topoisomerase (Top2) poisoning therapy is used to treat a broad range of cancers via induction of double strand breaks (DSBs) in cells undergoing replication and transcription. Preventing the repair of DSBs via inhibition of DNA-PK, an inhibitor of non-homologous end-joining (NHEJ), increases cell kill with Top2 poisons and has led to the initiation of several clinical trials. To elucidate the cellular mechanisms leading to synergistic activity of dual DNA-PK/Top2 inhibition we looked at their effects in cycling versus non-cycling cells, in 3D spheroids and in xenograft models. Combined DNA-PK/Top2 inhibition was found to not only increase the cell kill in proliferating cells, the cell population that is typically most vulnerable to Top2 poisoning, but also in non-proliferative but transcriptionally active cells. This effect was observed in both cancer and normal tissue models, killing more cells than high concentrations of etoposide alone. The combination treatment delayed tumor growth in mice compared to Top2 poisoning alone, but also led to increased toxicity. These findings demonstrate sensitization of Top2β-expressing, non-cycling cells to Top2 poisoning by DNA-PK inhibition. Expansion of the target cell population of Top2 poison treatment to include non-proliferating cells via combination with DNA damage repair inhibitors has implications for efficacy and toxicity of these combinations, including for inhibitors of DNA-PK currently in clinical trial.

## Introduction

Topoisomerase II (Top2) enzymes modulate the topology of DNA during replication, transcription, chromosomal segregation and DNA recombination. Features of DNA structures such as supercoiling, catenation and knotting make it poorly accessible to critical DNA processing enzymes. This is resolved by Top2 binding, which temporarily cuts the DNA helix, ‘untangling’ the complex structures^1^. The potential to convert these temporary breaks into permanent DSBs makes Top2 an attractive target for cancer therapy. Indeed, DNA topoisomerases are the molecular targets for several chemotherapy agents used to treat solid and hematological malignancies.

The mechanisms and structure–activity relationships of Top2-targeted anticancer agents was recently reviewed by Buzun et al*.*^2^. Top2 poisons, including the clinically active drugs etoposide and doxorubicin, bind to and stabilize the Top2-DNA covalent complex during replication and transcription and prevent the re-ligation of DNA broken ends. There are two Top2 isoforms, Top2α and Top2β, which share similar primary structure in humans. While both isoforms are necessary for chromosomal segregation and DNA recombination, Top2α is highly expressed in proliferating cells to guide DNA replication^3^ whilst Top2β has a more dominant role during transcription and is expressed ubiquitously^4^. When trapped by Top2 poisons, both isoforms generate DNA DSBs, however evidence has shown that the primary isoform target for Top2 poison-mediated cell cytotoxicity is Top2α, as Top2β knockdown/knockout cell lines show no significant effect on etoposide IC_50_^5^. Top2α is the primary molecular target for Top2 poison activity and Top2α expression levels are used as a prognostic biomarker for screening cancer patients suitable for Top2 poison treatment^6^. Thus, proliferating cells expressing Top2α are the most vulnerable to Top2 poison treatment, though DNA DSBs are also introduced in additional populations where other Top 2 isoforms are active.

The two primary DNA DSBs repair pathways are non-homologous end joining (NHEJ) and homologous recombination (HR). HR occurs during late S/G_2_ phases when sister chromatids are available as a sequence template for repair and consequently play a role in repair of replication-dependent DNA DSBs mediated by poisoning of the Top2α isoform. NHEJ occurs throughout the cell cycle and repairs DNA breaks by ligating the two broken DNA ends together. Overall, NHEJ is the predominant DNA DSB repair pathway in mammalian cells, with a particularly critical role for cells in non-proliferating stages of the cell cycle^7^. DNA-dependent protein kinase (DNA-PK), a member of the phosphatidylinositol 3-kinase-related kinase (PIKK) family, has been identified as a key component of the NHEJ pathway and is therefore an emerging target for therapeutic development^8^.

Recently, two DNA-PK inhibitors have shown sensitizing effects with Top2 poisons in preclinical models^9–12^ Both compounds have progressed to clinical trials: the Merck clinical candidate M3814 with etoposide and cisplatin (NCT03116971), and the Astra-Zeneca compound AZD7648 with pegylated liposomal doxorubicin (PLD) (NCT03907969). Pre-clinical efforts identified improved anti-cancer effects of the combination in vitro and in animal models without specifically investigating the target cell populations sensitized to Top2 poisons by the DNA-PK inhibitors, nor the mechanism for this sensitivity. Given the broad population of cells that are dependent on Top2 activity for facilitating access to DNA, all of which are targets for binding of Top2 poisons, it is plausible that inhibiting repair of the damage inflicted by Top2 poisons may result in toxicity for additional cell populations relative to the Top2α-targeting effects of Top2 poisons alone. Evidence from proteomics approaches has shown that both DNA-PK and Top2β are involved in regulating gene activation via binding to transcription factors^13–15^, providing an additional rationale for investigating the effects of the Top2 poisoning in combination with DNA-PK inhibition in cells undergoing transcription.

We hypothesized that DNA-PK plays a key role in repair of transcription-dependent DNA DSBs when Top2β is targeted by Top2 poisons. The impact of adding DNA-PK inhibitors would be sensitization of non-proliferative but transcriptionally active cells, extending the cell population killed via Top2 poison treatment. Using experimental models including cancer and normal tissue cell lines, 3D spheroids and mouse xenografts, we investigated the effects of Top2 poisons alone or in combination with DNA-PK inhibitors AZD7648 and M3814. Our results show that inhibition of DNA-PK results in enhancement of DNA damage and cell kill in non-proliferating but transcriptionally active cancer cells as well as normal tissue cells treated with Top2 poisons. Significant effects of the combination are seen on tumor growth, however dose-limiting toxicity was observed in mouse models, suggesting potential limitations for this combination.

## Material and methods

### Cell lines

HCT-116 (ATCC ID: CCL-247), A549 (ATCC ID: CCL-185), FaDu (ATCC ID: HTB-43), SiHa (ATCC ID: HTB-35), U87 (ATCC ID: HTB-14), and BT474 (ATCC ID: HTB-20) cells were obtained from ATCC. HCT-116 PRKDC^−/−^ was purchased from Thermo Fisher (Cat.NO A29442); HCT-116 BRCA2^−/−^ was obtained from Samuel Aparicio at BC Cancer Research Institute^16^; 48BR (CVCL-4T39) was a kind gift from Dr. Peggy Olive’s laboratory at BC Cancer Research Institute. All cell lines are maintained in MEM media (Gibco) supplemented with 10% fetal bovine serum (Gibco) and 1% (v/v). penicillin/streptomycin (Gibco). Cells were cultured at 37 °C in a humidified 5% CO_2_ incubator and passaged routinely when ~ 90% confluent.

### Chemicals

Etoposide was purchased from Teva (DIN: 02080036) and diluted in saline before use. Pegylated liposomal doxorubicin (PLD) was purchased from Taro (DIN: 02493020). AZD7648 (Cat. No HY-111783) and M3814 (Cat. No HY-101570) were purchased from MedChemExpress, and formulated in 1% hydroxypropyl methylcellulose/0.6% Tween80.

### Flow cytometry

Cell cycle progression and DNA damage were monitored in A549 cells after etoposide and DNA-PK inhibitor treatment at indicated times. Cells were fixed in 10% formalin (Fisher Scientific) in PBS for 15 min and permeabilized by 1% Triton X-100 (BioShop Canada Inc) in PBS at 4 °C for 30 min. The cells were then washed and incubated overnight at 4 °C in PBS containing 0.1% BSA (BioShop Canada Inc) and γH2AX antibodies (1:1000 dilution; EMD Millipore Corp, Cat No 05-636). Alexa Fluor 647 antibody (1:2000 dilution; Invitrogen, Catalog No A21245) was used to tag γH2AX antibodies and incubated at room temperature for 30 min. Cells were incubated in 50 μg/ml propidium iodide (Sigma-Aldrich) and 100 μg/ml RNase A (Invitrogen) for 30 min and 10^4^ cells per sample were then analyzed on a BD LSR FORTESSA (BD Bioscience) flow cytometer using 532 and 635 nm excitations. Fluorescent emissions were collected with 585/42 nm and 661/16 nm filters. Flowjo (BD Bioscience) software was used for data analysis.

### Clonogenic survival assay

Monolayer cells were harvested with 0.25% trypsin–EDTA (Gibco) and seeded at 4–8 × 10^3^ cells/well in 96 well plates. Cells were incubated with MEM media with or without 10% FBS for 24 h and then various doses of etoposide and DNA-PK inhibitors were used to treat either serum-starved or normal conditioned cells for 24 h. Treated cells were washed and harvested with trypsin–EDTA, then seeded into 96-well plates under three different dilutions (100 cells/well, 500 cells/well and 2500 cells/well). After incubation for 4 days, colonies were fixed in 1% formalin and stained with 10 µl/ml Hoechst 33342 (Sigma-Aldrich, Catalog No B2261) for 2 h. Images of each well were taken by a fluorescence microscope at 4× magnification and colonies were counted using Image J software (National Institute of Health) with custom defined algorithms.

### 3D spheroids cell culture

3D spheroids were grown in bulk using 500 mL Magna Flex spinner flasks (Wheaton) stirred constantly at 100 rpm. Growth took ~ 3 weeks to reach 400–500 µm diameter using an initial seeding number of 5 × 10^6^ cells. Spheroids were grown in MEM media with 5% FBS, 10 mM glucose (Gibco) and penicillin/streptomycin. Media was changed every 3–7 days during the culture period. Flasks were kept in a 37 °C incubator at 5% CO_2_.

### 3D spheroid 96-well assay

When spheroids reached a diameter of ~ 400 µm they were collected and dispensed into 2 mL tall polypropylene 96-well plates (Axygen). Settled spheroids were dispensed in 3–5 µL volumes into 1.2 mL of drug containing media to achieve 5–10 spheroids per well using a wide bore tip (Perkin Elmer, wide bore-P235) with an automated 96-channel liquid handler (PerkinElmer, Evolution P3). Multi-well plates were set up with desired drug dilutions prior to adding spheroids. Plates were then sealed with a silicone rubber mat (Axygen) in a glove box (Hypoxygen) at controlled gassing of 5% CO_2_, 20% O_2_. Upon sealing plates, they were then continuously rotated (20 rpm) with plates oriented on their sides and tilted so as to use the trapped air bubble to mix and maintain the spheroids in a free-falling state. Spheroids were exposed to etoposide ± inhibitors for up to 24 h prior to collection for cryo-sectioning or cell survival endpoints.

### 3D spheroid 96-well immunohistochemical endpoint

Following treatment as indicated, spheroids were incubated for 2 h with either 100 µM 5-ethynyl-2′-deoxyuridine (EdU) (Gold biotechnology) for replication or 1000 µM 5-ethynyluridine (EU) (Carbosyth China Ltd), for transcription prior to collection for cryo-sectioning and click-based staining. Spheroids were collected from all 96-wells simultaneously using a 96-channel pipetting head, allowed to settle and then dispensed as liquid-free piles on a stretched 13 × 13 cm latex sheet (Rite Dent) such that when relaxed the area contracted from the size of a 96-well plate (7.5 × 13 cm) down to a microscope slide (~ 2.5 × ~ 3.8 cm). The resulting spheroid micro-arrays were then immediately frozen via a – 30 °C aluminium block placed under the relaxed latex sheet and embedded in OCT sectioning medium (Fisher Scientific).

### 3D spheroid clonogenic endpoint

Following treatment as indicated, spheroids were collected and transferred to fresh drug-free media. Spheroids were washed three times with PBS prior to 20-min incubation with 0.25% trypsin–EDTA (Gibco) with continuous agitation at 37 °C. Following dissociation, cell suspensions were further diluted in fresh media with 10% FBS. Cell suspensions were then plated using either 6 cm tissue culture plates (Sarstedt, Germany) for experiments producing greater than 90% cell kill, or clear flat bottom 96-well polystyrene cell culture plates (Corning, Costar 3595) for low cell kill experiments. The 96-well colony plates were then left for 45 min prior to placement in 37 °C humidified incubator at 5% CO_2_ to allow settling of cells, otherwise non-uniform colony clumping in outer wells would occur. Following colony plating, the original cell suspensions were then counted in duplicate by diluting 25 µl aliquots from each well into additional flat-bottom clear polystyrene 96-well plates containing 25 µl of Hoechst 33342 (10 µl/ml final concentration) and propidium iodide (4 µl/ml final concentration). Cell suspensions were allowed to settle for 30 min prior to imaging using a fluorescence microscope for initial live/dead cell counting. Cell counts were then used to calculate the actual number of cells plated in individual wells. Counts were performed in replicates of 2 to ensure reproducibility. 96-well plates were incubated for 4 days at which point they were fixed in 1% formalin and stained with Hoechst 33342 for 2 h and images of each well were then taken by fluorescence microscope at 4× magnification and colonies were counted using Image J software with custom defined algorithms. 6 cm plates were incubated for 12 days to allow colony formation and were then stained using 2 g/L malachite green (Sigma-Aldrich) in water and counted manually.

### In vivo studies

All studies involving mice were performed at BC Cancer Research Institute Animal Resource Centre and were approved by the University of British Columbia Institutional Animal Care and Use Committee under approved animal study protocol A21-0059. All animals were treated in accordance with the Canadian Council on Animal Care ethical guidelines and are reported in accordance with ARRIVE 2.0 guidelines. Rag2M mice were originally purchased from the Taconic Biosciences.

For A549 tumor growth delay studies, 10^6^ A549 cells were inoculated in the gastrocnemius muscle of 61 10–13-week-old Rag2M male mice in a volume of 50 μL, including an overage of 20% to account for variable tumor growth rates. When the median tumor volume of the cohort exceeded 150 mm^3^ animals were stratified based on tumor volume ranges; animals bearing the smallest tumor volumes were excluded from the study (n = 13). Remaining animals (cohort size of n = 48, 30 for etoposide tumor growth delay and 18 for PLD growth delay) all had tumor volumes within the volume range of 160–225 mm^3^. Animals were assigned to treatment groups using stratified randomization and a random number generator to ensure a range of tumor volumes was assigned to each group. No power calculations were performed a priori. A sample group size of n = 6 was determined appropriate for this study due to prior observations of consistent tumor response to chemotherapy and sensitization responses by DNA damage repair inhibitors, without numerous outliers or abnormalities. Significant morbidities were observed that required humane termination endpoints due to significant weight loss in particular treatment groups; larger group sample sizes would not have improved this result.

Etoposide was administered intraperitoneally (ip) at 5 mg/kg on days 0, 1 and 2 of each week for 3 weeks. Pegylated liposomal doxorubicin (PLD) was administered by intravenous (iv) injection at 6 mg/kg once per week for 3 weeks. AZD7648 was administered via oral gavage (po) at 10, 30 or 100 mg/kg at the same times in combination with Top2 poisons. For control groups, an equivalent volume of drug vehicle was administered. Tumor size and mice weights were monitored 3 times per week, with tumor volumes measured by calipers and calculated using formula V = π/6 (L × H × W). Measurements were done via a blinded recorder. Mice were euthanized at experimental end point, when tumor volumes reached 1000 mm^3^, or at humane end point, including animals exceeding > 20% loss from pre- treatment weights. Animals that had to be euthanized due to humane endpoint are reported in the study results. All animals were maintained in the same housing and husbandry conditions, with dough diet supplements provided to all experimental mice during the treatment period.

For immunohistochemistry-based studies, a total of 18 mice were used. No power calculations were performed a priori. A sample size of n = 6 was determined appropriate for this study due to prior observations of consistent tumor response using the EdU assay, without numerous outliers or abnormalities. Whole cryosections of tumor samples are analyzed to create a robust data set. 10^6^ FaDu cells were injected in the gastrocnemius of 13 week-old Rag2M male mice and grown to a minimum of ~ 300 mm^3^ in volume. Etoposide (40 mg/kg ip) ± AZD7648 (30 mg/kg po) were administered 24 h before tumor harvest. At 2 h before tissue collection, EdU was administered (60 mg/kg ip). After excision, tumors were cooled to – 20 °C on an aluminum block and covered in OCT embedding medium (Tissue-TEK OCT).

### Immunohistochemical staining

Tumor or spheroid cryosections (10 µm thick) were cut with a Cryostar HM560 (Microm International GmbH) cryostat, fixed immediately in 10% phosphate buffered formalin for 15 min and then transferred to PBS + 0.1% Tween 20 (PBST). Vasculature was stained using a CD31 antibody (1:500 dilution; BD Pharmingen, Catalog No 553370) for 1 h, followed by 1:500 Alexa-anti-rat 488 nm secondary (Invitrogen, Catalog No A11006) for 30 min. DNA damage was stained using 1:500 of p53BP1 antibody (Cell Signaling, Catalog No 2675S) for 1 h, followed by 1:500 Alexa-anti-rat 647nm secondary (Invitrogen, Catalog No A21472) for 30 min. Top2 was stained using 1:500 of Top2α antibody (Novus, Catalog No NBP2-53281) or Top2β antibody (Novus, Catalog Bo NBP1-89527) for 1 h, followed by 1:500 of Alexa-anti-rabbit 488 nm secondary (Invitrogen, Catalog No A11034) for 30 min. For EdU and EU detection, slides were re-fixed in 10% formalin for 10 min, prior to EdU/EU staining using click chemistry cocktail: L-ascorbic acid (100 mM), CuSO_4_ (4 mM) and Sulfo-Cy3-Azide (5 µM) (Sigma-Aldrich) along with the nuclear marker Hoechst 33342 (20 µg/ml) for 30 min. All dilutions were made in PBST. Slides were then washed for 30 min and mounted with PBS prior to imaging.

### Image acquisition and data analysis

Slides were loaded onto a custom-built imaging system a Nikon Plan Fluorite Imaging 10× objective (Nikon), a cooled PCO Edge 4.2 sCMOS camera (PCO), run using customized algorithms in ImageJ software (ImageJ, https://imagej.nih.gov/ij/). Using this system, images of entire microscope slides were captured at a resolution of 0.65 µm/pixel.

Using the ImageJ software application and user supplied algorithms, images of CD31 fluorescence, 53BP1, EdU and Hoechst 33342 from each tumor section were overlaid, tumor boundaries described, and areas of necrosis and staining artefacts were removed (folds, tears, debris, etc.). Objects positive for CD31 were then identified on the CD31 image layer using a threshold determined to be 10 standard deviations above the tissue background levels. CD31 objects that were less than 10 µm^2^ in size were considered artefacts and removed from the analysis. Analysis was then carried out to measure the distance from each point in the tissue to the nearest CD31 positive object noting its intensity to then determine the relation between proliferation or 53BP1 signal as a function of the distance to the nearest blood vessel. The data were tabulated so as to determine the profile of EdU and 53BP1 staining versus distance to blood vessels, or the average intensity of pixels is reported for p53BP1. A similar approach was taken for spheroid analysis, using the outer spheroid edge as the reference point rather than the CD31 objects.

### Statistical analysis

All statistical analyses were performed using Graphpad Prism software (version 9.3). Drug dose response survival curves were fitted with non-linear regression curve fitting tools in Prism, using the “Absolute IC_50_, X is log (concentration)” model. Survival summary and etoposide pIC_50_ bar graphs were assessed via unpaired, two tailed t-tests. Time to tumor volume tripling was assessed for all groups by ANOVA followed by Turkey multiple comparisons tests. Mouse percentage weight loss at day 7/14 analyses were assessed by ANOVA Kruskal–Wallis test. Tumor p53BP1 signal intensity analyses were performed via unpaired two tailed t-tests. Spheroid p53BP1 signal intensity analyses were via ANOVA followed by Tukey multiple comparisons tests. Calculated p-values are reported as *p $\le$ 0.05; **p $\le$ 0.01; ***p $\le$ 0.005; ****p $\le$ 0.001.

## Results

### Abrogation of the NHEJ repair pathway but not HR sensitizes 3D tumor spheroids to etoposide

**Figure 1:**
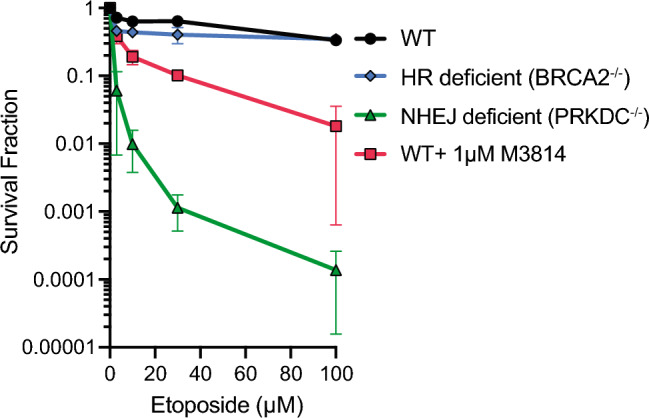
Abrogation of the NHEJ repair pathway but not HR sensitizes 3D tumor spheroids to etoposide, which saturates at 50% cell kill. Clonogenic surviving fraction ± SD of HCT-116 wild-type, BRCA2^−/−^, and PRKDC^−/−^ spheroids treated with etoposide (0, 3, 10, 30, 100 µM) for 24 h.

3D spheroid tissue models re-capitulate the oxygen and nutrient gradients of the solid tumor microenvironment to result in a mixed cell population containing a greater proportion of non-proliferating quiescent cells relative to monolayer culture, where proliferating cells are more likely to dominate response to etoposide treatment^17^. In the 3D spheroid model, treatment with etoposide has a dose–response relationship that saturates beyond about 10 µM, with approximately 40% of cells surviving even at 10× higher doses (Fig. 1). To elucidate the roles of HR and NHEJ in sensitizing the spheroids to Top2 poison induced DNA damage, we performed clonogenic survival assays on HCT-116 spheroids that were either deficient in HR (BRCA2^−/−^) or NHEJ (PRKDC^−/−^) following etoposide exposure. After 24 h, HR deficient spheroids show a similar etoposide sensitivity profile relative to wild-type HCT-116 spheroids, with approximately 60% cell kill, consistent with selective targeting of the s-phase fraction within spheroids^18^, while NHEJ-deficient spheroids experienced ~ 4 logs additional cell kill. The addition of 1 µM DNA-PK inhibitor M3814 increased the cell kill in wild-type spheroids by a factor of 10 compared to etoposide treatment alone. These results indicate that NHEJ is the main repair pathway for etoposide induced DNA damage and DNA-PK inhibition can effectively sensitize cell spheroids beyond what increasing concentrations of etoposide alone is able to achieve.

### DNA-PK inhibitor sensitizes non-proliferating cells to etoposide treatment in monolayer culture

**Figure 2:**
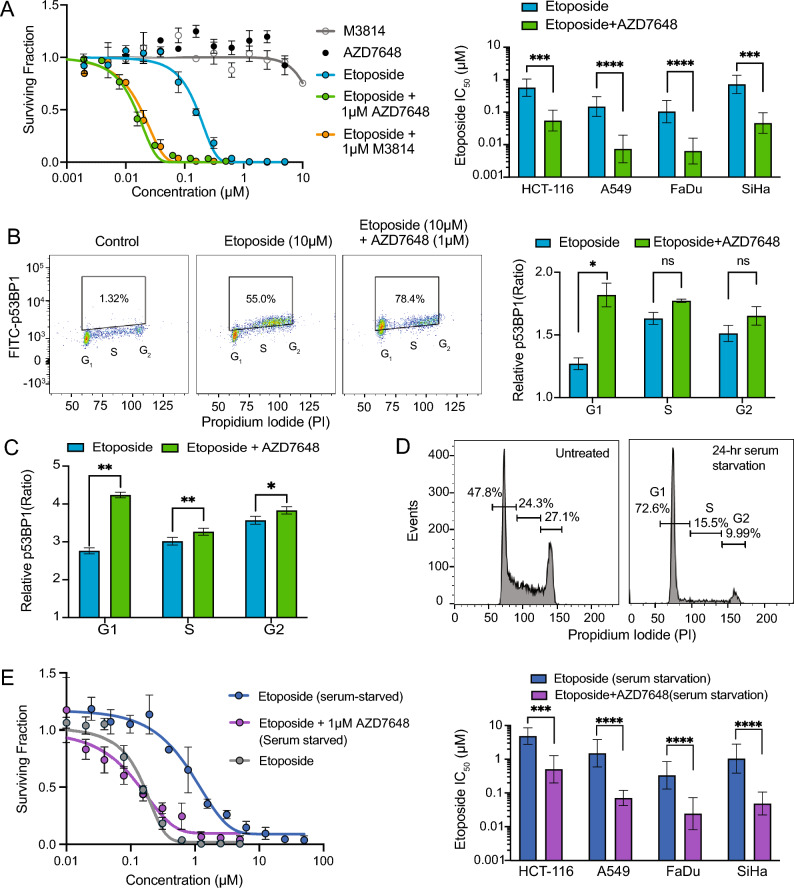
DNA-PK inhibitors sensitize monolayer cell cultures to etoposide treatment. (**A**) Clonogenic survival of cancer cell lines treated with etoposide ± 1 µM AZD7648 or 1 µM M3814 for 24 h (mean ± SD). Survival curve of FaDu cells, AZD7648 and M3814 given alone are shown (left panel). IC_50_ ± SD of HCT-116, A549, FaDu and SiHa cell lines treated with etoposide ± 1 µM AZD7648 for 24 h (right panel). Experiments performed at least 3 times. Addition of AZD7648 significantly decreased etoposide IC_50_ (HCT-116: p ≤ 0.005, A549: p ≤ 0.0001, FaDu: p ≤ 0.0001, SiHa: p ≤ 0.005). (**B**) Evaluation of A549 cells’ DNA damage and cell cycle by p53BP1 and propidium iodide (PI) dual staining assay after 6 h treatment of 10 µM etoposide ± 1 µM of AZD7648 or vehicle. The experiments were carried out at least 3 times. Representative counter plots of A549 cells in control, etoposide alone, and etoposide + AZD7648 treatment conditions (left panel). The number (box) represents the percentage of total cells. Cell cycle-specific relative p53BP1 immunofluorescent ratio of etoposide and etoposide + AZD7648 groups over a control group (right panel). The difference is only statistically significant in the G_1_ population (p ≤ 0.01). (**C**) Evaluation of 48BR cells’ DNA damage and cell cycle by p53BP1 and propidium iodide (PI) dual staining assay after 4 h treatment of 10 µM etoposide ± 1 µM of AZD7648 or vehicle. The experiments were carried out at least three times. Cell cycle-specific relative p53BP1 immunofluorescent ratio of etoposide and etoposide + AZD7648 groups are shown over a control group. The differences are statistically significant for all phases, with the largest magnitude change in G_1_ (G_1_: p ≤ 0.005; S: p ≤ 0.01; G_2_: p ≤ 0.05). (**D**) Analysis of FaDu cell cycle distribution using propidium iodide after 24 h of serum starvation. Numbers (over gate bars) represent the % of total cells. (**E**) Clonogenic surviving fraction ± SD of cancer cell lines treated with etoposide ± 1 µM AZD7648 under serum starved conditions for 24 h. Survival curve of serum starved FaDu cells under etoposide/etoposide + AZD7648 treatment. Etoposide alone treatment under normal (non-serum starved) condition was also shown (left panel). IC_50_ ± SD of HCT-116, A549, FaDu and SiHa cell lines treated with etoposide ± 1 µM AZD7648 under serum starvation for 24 h (right panel). The IC_50_ differences for etoposide vs etoposide + AZD7648 groups are statistically significant (HCT-116: p ≤ 0.005, A549: p ≤ 0.0001, FaDu: p ≤ 0.0001, SiHa: p ≤ 0.0001).

The effect of DNA-PK inhibition in combination with Top2 poisons was assessed via clonogenic survival assays performed on monolayer cell cultures treated with etoposide ± DNA-PK inhibitors AZD7648 or M3814. Dose response curves for FaDu cells (Fig. 2A left panel) show monotherapy treatment with AZD7648 or M3814 had no effect on survival up to 10 µM, however 1 µM of either DNA-PK inhibitor in combination with etoposide showed a significant reduction in cell survival relative to etoposide alone. These data were used to calculate etoposide IC_50_ values shown in Fig. 2A (right panel) for FaDu and additional cell lines, with values consistently lower for 1 µM AZD7648 + etoposide relative to the Top2 poison alone, indicating that DNA-PK inhibitors sensitize monolayer tumor cells to etoposide by at least a factor of 10.

The amount of DNA damage sustained by cells in G_1_, S and G_2_ stages of the cell cycle was assessed using flow cytometry based on propidium iodide (PI) staining and analysis of p53BP1 staining following treatment with etoposide ± DNA-PK inhibitor AZD7648. Results in A549 cells (Fig. 2B) show an increase in p53BP1 signal intensity for cells co-treated with etoposide + AZD7648 (78.4% of cells are positive) relative to etoposide alone (55% of cells are positive). Cell cycle-specific analysis indicates a significant increase in DNA damage for the combination relative to etoposide alone for the G_1_ cell population, with smaller changes in the proliferating S and G_2_ cell populations. Similar results are seen with 48BR (human skin fibroblasts) cells where these normal cells sustained significantly more DNA damage when treated with etoposide + AZD7648 compared to etoposide alone, with the largest effects in G_1_ phase (Fig. 2C).

The impact of etoposide ± DNA-PK inhibition on non-proliferating cells was assessed using a clonogenic survival assay on serum-starved cells. Monolayer FaDu, SiHa, HCT-116 and A549 cells were incubated in serum-free media for 24h to result in over 70% of cells residing in G_0/1_ phase (FaDu cell line shown in Fig. 2D). Clonogenic survival assays were then performed on both serum-starved and non-starved cells to generate dose–response curves for etoposide (2.5 nM–50 µM) ± 1 µM AZD7648 for 24 h. Results show that serum-starved cells are more resistant to etoposide treatment than the non-starved cells, however the addition of DNA-PK inhibitor AZD7648 re-sensitized the serum-starved cells (Fig. 2E left panel) to a similar degree as was seen in the non-serum starved cells (Fig. 2A left panel). These results were repeated in multiple cell lines, where the IC_50_ value of etoposide alone is consistently much higher than etoposide in combination with AZD7648 (Fig. 2E right panel).

### DNA-PK inhibitors sensitize non-proliferating, transcriptionally active cells in 3D tumor spheroids to etoposide

**Figure 3:**
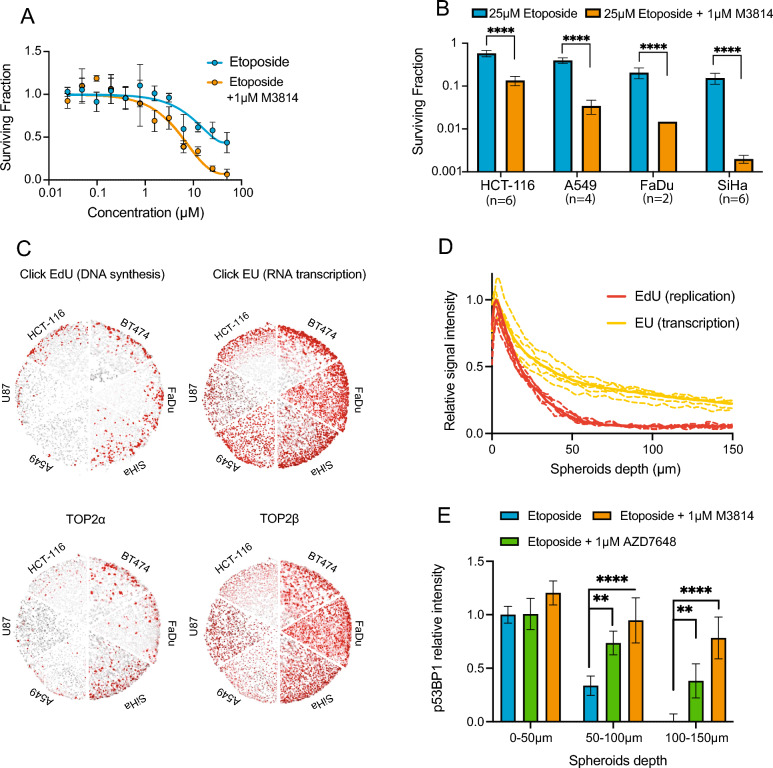
DNA-PK inhibitors sensitize 3D tumor cell spheroids to etoposide treatment. (**A**) Clonogenic survival of FaDu cell spheroids treated with etoposide ± 1 µM M3814 for 24 h (mean ± SD). (**B**) Survival fraction of HCT-116, A549, FaDu, and SiHa spheroids treated with 25 µM etoposide + 1 µM M3814 for 24 h (mean ± SD) (p ≤ 0.0001 for all cell lines). Experiments were performed at least 3 times except for FaDu cells. (**C**) Representative images of EdU, EU, Top2α, and Top2β (red) staining in HCT-116, A549, FaDu, SiHa, U87 and BT474 tumor cell spheroids. Cell nuclei were stained using Hoechst 33342 (grey). (**D**) Evaluation of EdU and EU signal intensity versus cell depth in untreated HCT-116 spheroids. Dashed lines represent individual experiment repeats while solid lines represent group averages. (**E**) Analysis of p53BP1 signal intensity (mean ± SD) at different depths into tumor cell spheroids (0–50 µm, 50–100 µm and 100–150 µm). HCT-116 spheroids were treated with 25 µM of etoposide in the presence of 1 µM M3814, 1 µM AZD7648 or vehicle for 6 h before cryosectioning and staining. Addition of M3814 and AZD7648 both significantly increased p53BP1 intensity in 50–100 µm and 100–150 µm layers of spheroids (p ≤ 0.01 for AZD7648, p ≤ 0.001 for M3814).

To further explore the sensitization of cells to Top2 poisons by DNA-PK inhibition, we evaluated this combination therapy in tumor spheroids. As described above, 3D tissue spheroid models contain a greater proportion of non-proliferating, quiescent cells relative to monolayer culture. Figure 3A shows a dose–response survival curve of FaDu spheroids to etoposide, indicating the spheroids are highly resistant to etoposide compared to monolayer culture, however addition of 1 µM M3814 shows sensitization beyond the maximum effects seen with etoposide alone. Similar results are shown in additional cell lines HCT-116, SiHa, and A549, where the surviving fraction of spheroids decreased consistently in the combination treatment groups of 25 µM etoposide + 1 µM M3814 relative to 25 µM etoposide alone.

To investigate the potential contribution of transcription to the response of dual DNA-PK/Top2 inhibition, immunohistochemical staining of transcription and replication status was assessed as a function of depth into the 3D spheroids and these results were compared along with Top2α and Top2β staining profiles. DNA replication and RNA synthesis patterns were visualized via ‘click’ chemistry staining with EdU and EU respectively^19,20^. Our results showed that both Top2α and EdU staining were restricted to the edge of spheroids (< 50 µm) while Top2β and EU staining were expressed more ubiquitously, extending into deeper layers of spheroids (Fig. 3C,D). DNA damage was also assessed using p53BP1 in spheroids treated with etoposide ± DNA-PK inhibition. When etoposide was exposed to spheroids alone, p53BP1 staining was only detected in the first 50 µm of the spheroids, consistent with greater DNA damage occurring in the proliferating fraction of spheroid cell populations. However, when 1 µM AZD7648 or M3814 was added, the p53BP1 was extended further into the spheroids (50–150 µm) where DNA synthesis was reduced or absent, but where RNA synthesis was still active (Fig. 3E).

### DNA-PK inhibition is synergistic with Top2 poisoning in vivo*:* resulting in delayed tumor xenograft growth but also dose-limiting toxicity

**Figure 4:**
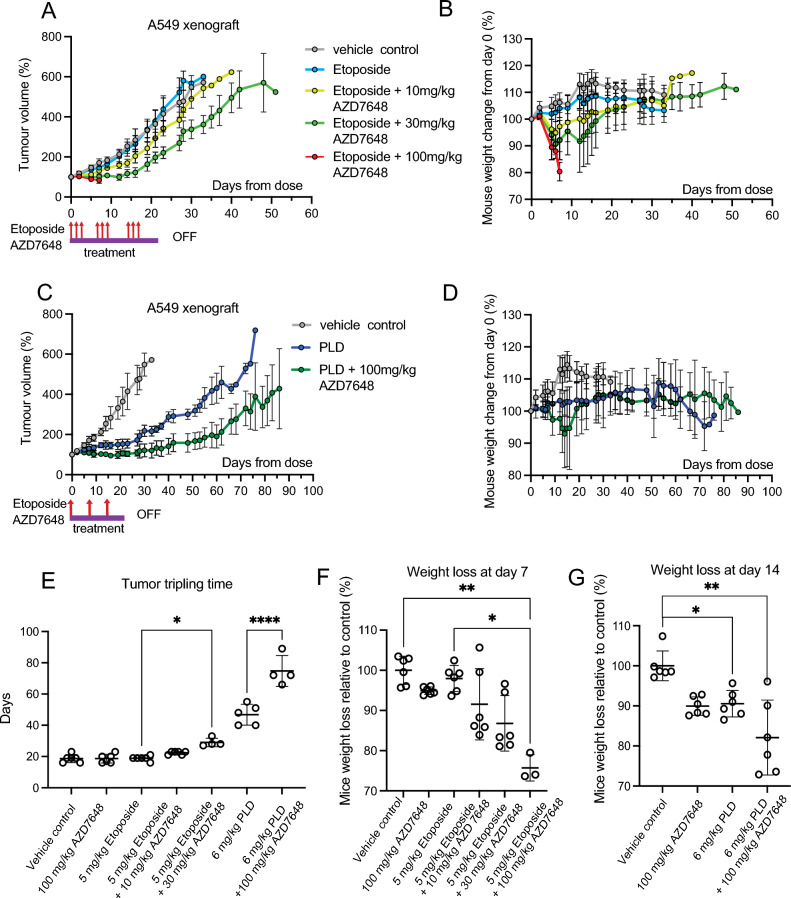
DNA-PK inhibitor AZD7648 administered in combination with Top2 poisons delays tumor growth. (**A**) and (**C**), relative tumor volumes (mean ± SD) of A549 tumor growth plotted vs. time. Tumor bearing mice were randomized (n = 6 per group) at a mean tumor volume of 185mm3 (SD = 17.6) and 192 mm3 (SD = 17.6) respectively. For etoposide treatment (**A**), 5 mg/kg etoposide was administered intraperitoneally, and AZD7648 was administered orally at doses of 10, 30, and 100 mg/kg. For PLD treatment (**B**), 6 mg/kg of PLD was administered intravenously with or without 100 mg/kg AZD7648 administered orally. All treatments lasted for 3 weeks. (**B**) and (**D**) show relative mice weight change (mean ± SD) of A549 tumor-bearing animals after treatments with etoposide and PLD respectively, plotted against time. (**E**) Tumor tripling time for mice shown in (**A**) and (**C**); 100 mg/kg AZD7648 alone treatment was done in a separate study. Dots represent the individual mice in each group. There is a significant increase in tumor tripling time for etoposide + 30 mg/kg AZD7648 (p ≤ 0.05) relative to etoposide alone, as well as between PLD vs PLD + 100 mg/kg AZD7648 (p ≤ 0.001). (**F**) Percentage of mouse weight loss relative to control (mean ± SD) at day 7 for etoposide treatment. The difference between vehicle control vs 5 mg/kg etoposide + 100 mg/kg AZD7648 and 5 mg/kg etoposide vs 5 mg/kg etoposide + 100 mg/kg AZD7648 groups are statistically significant (p ≤ 0.01 and p ≤ 0.05 respectively). (**G**) Percentage of mouse weight loss relative to control (mean ± SD) at day 14 for PLD treatment. The difference between vehicle control vs 6 mg/kg PLD and 6 mg/kg PLD vs 6 mg/kg PLD + 100 mg/kg AZD7648 groups are statistically significant (p ≤ 0.01 and p ≤ 0.05 respectively).

We next tested Top2 poisons ± DNA-PK inhibition therapy in mice bearing FaDu or A549 xenografts. Figure 4 shows the anti-tumor effect of DNA-PK inhibition via AZD7648 with the Top2 poisons etoposide or pegylated liposomal-doxorubicin (PLD) in A549 xenografts. The AZD7648 treatment alone had no anti-tumor effect (Fig. 4E). The relatively low dose of etoposide (5 mg/kg 3×/week for 3 weeks) had little antitumor effect compared to vehicle controls, and adding the lowest dose of AZD7648 (10 mg/kg 3×/week for 3 weeks) produced no sensitization. However, a significant delay in time to 3× tumor volume was observed at a higher dose of AZD7648 (30 mg/kg 3×/week for 3 weeks) with etoposide, extending to 28 days relative to 19 days for etoposide alone (Fig. 4E). Further increasing the AZD7648 dose to 100 mg/kg could not be completed due to severe normal tissue toxicity requiring an early endpoint termination (7 days after initial treatment). Weight loss of mice in the combination treatment groups are seen to follow a similar dose-dependent pattern to the growth inhibition effects, with all the groups experiencing rapid weight loss, and the highest combination group of 100 mg/kg AZD7648 group exceeding 20% in the first week (Fig. 4B,F). This is consistent with the sensitivity of normal tissue cells relative to cancer cells shown with monolayer cultures (Fig. 2C).

A549 tumor-bearing animals treated with 6 mg/kg PLD once per week for 3 weeks as a single agent had a significant tumor growth delay relative to controls with time to tumor volume tripling extended from 19 to 48 days (Fig. 4E). The addition of 100 mg/kg AZD7648 to PLD further delayed the tumor growth to 72 days relative to 48 days for PLD as a single agent (Fig. 4E). Moderate normal tissue toxicity was seen for PLD alone as well as the combination group, as all mice had to be provided with dough diet supplements in order to not exceed 20% weight loss in the treatment groups; control groups gained weight with the dough diet (Fig. 4D,G). Similar results are also seen in FaDu xenografts (data not shown).

### AZD7648 enhances DNA damage in FaDu xenograft tumors treated with etoposide in both cycling and non-cycling cell populations

**Figure 5:**
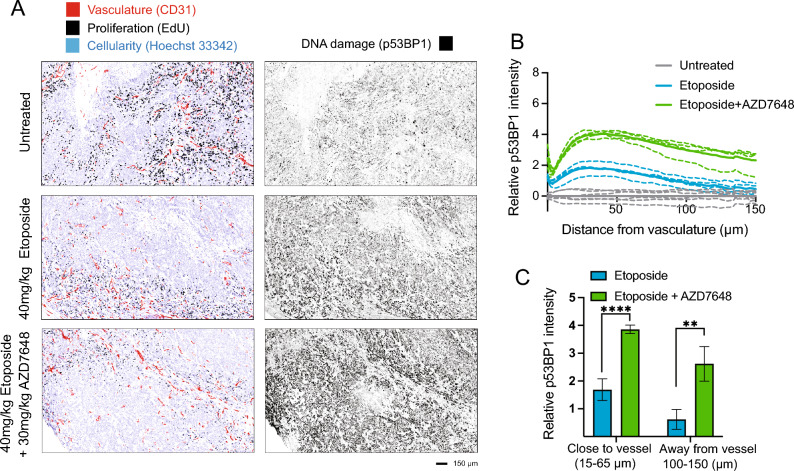
The DNA-PK inhibitor AZD7648 enhances DNA damages induced in xenograft tumors treated with Top2 poison etoposide. (**A**) Representative images of EdU, CD31, and p53BP1 IHC staining of FaDu xenograft tumors treated with 40 mg/kg etoposide (ip) as single agent or in combination with 30 mg/kg AZD7648 (po). At 4 h animals were further dosed with 60 mg/kg EdU (ip), with tissues collected at 6 h after the chemotherapy treatment. (**B**) Evaluation of p53BP1 signal intensity as a function of distance from blood vessels (CD31). Controls, etoposide and etoposide + AZD7648 groups are indicated by grey, blue, and green lines respectively. Solid line represents the average of all experiments while dashed lines represents individual repeats. (**C**) Quantitative analysis comparing p53BP1 signal intensity in areas close to blood vessels (15–65 µm) and farther away from blood vessels (100–150 µm) between etoposide alone and etoposide + AZD7648 groups shows a statistically significant result (p ≤ 0.0001 for 15–65 µm, p ≤ 0.005 for 100–150 µm).

We performed immunohistochemical staining and analysis of FaDu xenograft tumors treated with etoposide ± AZD7648 to examine the location of DNA damage and proliferating cells relative to tumor vasculature. Single agent treatment with a relatively high dose of 40 mg/kg etoposide significantly induced DNA damage in FaDu tumors at 6h. Staining for p53BP1 was detected primarily in regions where functional blood vessels (CD31) and proliferating cells (EdU) are located (representative images shown in Fig. 5A). The addition of 30 mg/kg AZD7648 extended the region of cells with positive DNA damage, as p53BP1 staining is seen to be more intense, and is now also detected in regions with fewer blood vessels.

Our results showed the EdU signal of control tumors peaking close to blood vessels (< 50 µm) and gradually decreasing as the distance increases (Fig. 5A). This is consistent with features of the solid tumor microenvironment where cells close to blood vessels are proliferative while those farther away become non-proliferative and quiescent^21–23^. Quantitative analysis was done to map the p53BP1 staining intensity as a function of distance to nearest CD31-stained blood vessels. Staining of p53BP1 in drug-treated tumors increases to a peak close to blood vessels in treated groups, before decreasing again farther away. However, while the etoposide alone group shows p53BP1 staining returning to control levels at distances far from vessels, addition of the DNA-PK inhibitor AZD7648 results in greater levels of persistent p53BP1-labeled DNA damage (Fig. 5B). Significant p53BP1 intensity increases were observed between etoposide single treatment and combination groups for regions ‘close to blood vessels’ and ‘away from blood vessels’ (p $\le$ 0.0001 and p $\le$ 0.005 respectively) (Fig. 5C). These findings suggest that adding DNA-PK inhibitors sensitizes non-proliferative tumor cells to etoposide treatment in vivo.

Overall, we have demonstrated that DNA-PK inhibitors sensitize cells to Top2 poison treatment in 3 pre-clinical models: cell culture, 3D spheroids and tumor-bearing mice. Relative to Top2 inhibition alone, immunohistochemical staining and flow cytometry analysis indicate enhanced DNA damage in non-proliferating and G_1_ phase cancer and normal tissue cells in treatments when combining etoposide and DNA-PK inhibitors. Clonogenic survival assays and in vivo tumor growth delay studies confirm that the observed increase in DNA damage translates to greater cell kill and longer tumor growth delay in combination treatments compared to Top2 poison alone, however dose-limiting toxicities were also observed. Together, these data demonstrate that combining DNA-PK inhibitors with Top2 poisons is an effective treatment approach that is able to kill non-proliferating, quiescent tumor cells that are otherwise resistant to Top2 poison treatments to result in greater anti-cancer effects. However, the observed concomitant increase in toxicity to non-cancerous cells suggests a need for targeting the cell-killing effects to tumor tissues.

## Discussion

We have shown that DNA-PK inhibitors such as AZD7648 enhance the anti-cancer effects of Top2 poisons by expanding the population of targeted cells to include the non-proliferating, but transcriptionally active cells prevalent in the tumor microenvironment.

Top2 poison cytotoxicity is mediated by trapping Top2 proteins on the broken ends of DNA, with DNA DSBs generated as the protein-DNA complex collides with the machinery of replication or transcription. Evidence has shown that Top2α is the major isoform expressed in proliferating cells to guide replication, while Top2β is expressed in all cell types to guide transcription^24,25^. Both isoforms can generate DNA DSBs when trapped by Top2 poisons, though Top2α mediated replication-dependent mechanisms seem to be the main cause of Top2 poison cytotoxicity^26,27^. Our DNA damage and cell survival results are consistent with these patterns. Cell culture and 3D spheroid models showed Top2α is mostly expressed in proliferating cells and treatment with etoposide increased DNA damage in this population, while the non-proliferating fraction is 10 times more resistant. Further, data from spheroids shows a dose–response relationship for etoposide cell-killing effect that plateaus with a significant > 40% surviving fraction even when the concentration is increased 10 times, suggesting a significant population of resistant cells (Figs. 1, 3).

**Figure 6:**
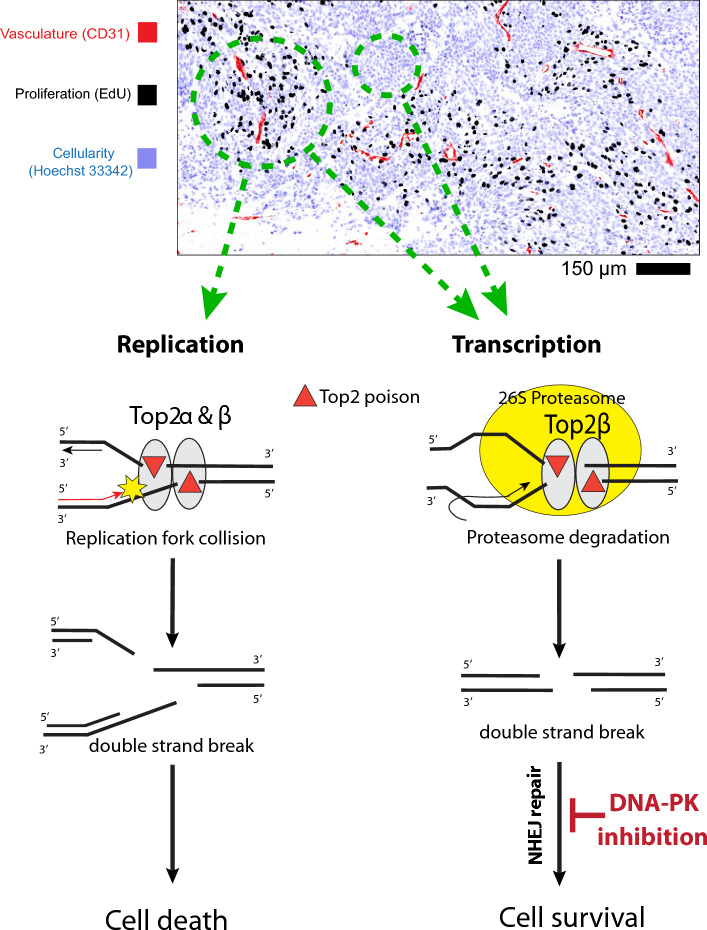
Tumor derived quiescence leads to a population of Top2 poison resistant cells which can be targeted by DNA-PK inhibition. During replication, trapped Top2 is removed by replication run-off, leaving long single strand overhangs at the DNA 3′ end, which leads to classical Top2 poisoning-driven cellular death. During transcription, trapped Top2 is first degraded by 26S proteasome followed by Tdp2 cleavage, leaving DNA DSBs with two blunt ends that can be efficiently repaired by NHEJ. Inhibition of DNA-PK blocks the NHEJ pathway, leading to a novel mode of Top2-poison-derived cellular death.

Top2β is the other Top2 isoform that is expressed in both proliferating and non-proliferating cells and mainly participates in transcription, as is confirmed in our spheroid data. The two isoforms share the same catalytic mechanism and are equally targeted by Top2 poisons^28,29^, however repair of the resulting DSBs is cell cycle-specific, with NHEJ involved in all phases of cell cycles and HR only activated during late S to G_2_ phase. Our data again confirms what others have shown, that proliferating cells have greater sensitivity to Top2 poisons relative to non-proliferating cells with the implication that proliferating cells are less capable of repairing Top2 poison-related damage. Solid cancers contain a mixed population of proliferating and non-proliferating cells due to the complexity of the microenvironmental gradients of oxygen and nutrient supply, such that non-proliferating tumor cells distal to functional blood vessels are resistant to Top2α-targeted anti-cancer effects^30^. HR is a relatively slow but accurate process and a high dose of Top2 poison can induce DNA DSBs that can exceed capacity for HR repair^31^. The DSBs induced during transcription, on the other hand, may be more readily repaired via NHEJ, a repair process that is faster and has a larger capacity compared to HR^32^. Consequently, non-proliferative cells have greater opportunity to repair the DNA damage induced by Top2 poisons through NHEJ repair. Evidence from proteomic approaches further supports this hypothesis by showing that trapped Top2 is removed from DNA broken ends by different mechanisms during replication and transcription^33–35^. This results in different forms of DNA DSBs at 3′ end between replication and transcription (long vs short 3′ ssDNA overhangs) which favors HR and NHEJ repair respectively. Figure 6 summarizes the cellular responses to trapped Top2 for cells undergoing replication or transcription with their subsequent cell fates^36^.

Our data provides evidence to support the argument that NHEJ is the critical repair pathway for non-proliferating cells to repair the DNA DSBs introduced by Top2 poisoning treatment. Blocking the NHEJ pathway with the DNA-PK inhibitor AZD7648 sensitizes tumor cells to etoposide and doxorubicin, with the most significant impact on non-proliferating cells. Staining data in spheroids shows that Top2β is expressed ubiquitously in both proliferating and non-proliferating cells mapped via incorporation of EdU overlapping with the transcription profile of spheroids mapped using incorporation of EU. Addition of DNA-PK inhibitors enhanced the DNA damage of both proliferating and non-proliferative but transcriptionally active cells. This greater DNA damage translated to greater cell kill in vitro and increased anti-tumor effects in vivo, as demonstrated by the outcome of combining the DNA-PK inhibitor with Top2 poisons in clonogenic survival and tumor growth delay assays. Overall, our evidence suggests that Top2 poisons are most effective at killing cells unable to repair the damage, which appears to preferentially be the HR-dependent cells in S-phase. Quiescent cells that are able to use NHEJ to repair the DNA DSBs occurring during transcription by Top2β poisoning are less likely to die, however this can be overcome with inhibition of the key NHEJ protein, DNA-PK. Similarly, inhibiting other components of NHEJ pathway was also found to increase the cytotoxicity of Top2 poisons. Srivastava et al*.* has shown that a ligase IV inhibitor, SCR7, inhibits NHEJ pathway, and confers greater tumor control when treated in combination with etoposide. However, the detailed molecular mechanism resulting in the cytotoxicity and target cell populations was not investigated^37^.

Despite the significant tumor growth delays achieved in our in vivo studies, the adverse effects in the combination treatment groups was evident. As most post-mitotic normal tissues are also non-proliferative but transcriptionally active, this combination therapy may result in a narrowing of the therapeutic index for Top2 poisons, limiting the range of doses where Top2 poisons can be effective without unacceptable adverse effects. Moderate to severe toxicities were observed in our combination treatment groups, where the highest dose of AZD7648 in combination with normally well tolerated 5 mg/kg etoposide induced excessive weight loss during the first week of treatment, requiring humane termination of animals in the study. The weight loss could be the result of excessive toxicity burden on the intestines, a highly proliferative organ with high sensitivity to toxic chemotherapy. Pegylated liposomal doxorubicin (PLD) has been found to have greater tumor-targeting specificity owing to a longer plasma half-life with lower peak concentrations^38^. Toxicity in the combination treatment groups still occurred with PLD, but was significantly less than that seen with etoposide. Other groups combining Top2 poisons with DNA-PK inhibitors have reported similar anti-cancer efficacy results, however they also report toxicity, with 15%^12^ and 10%^11^ body weight loss for animals treated with PLD (2.5 mg/kg) + AZD7648 (37.5 mg/kg) relative to PLD alone. However, this toxicity can be overcome when a more localized chemotherapy is applied, such as using chemotherapy drug-loaded DC M1 polymer beads^39^. Other methods to limit toxicity and establish a therapeutic ratio in the clinic involve careful dosing and treatment scheduling design, optimizing cancer cell kill while minimizing dose-limiting toxicities. Mathematical models that incorporate cell cycle, DNA damage and repair kinetics have been used to predict in vivo xenograft response and generate tolerable treatment dosing schedules, as has been used in the phase I clinical trial design for ATR inhibitor AZD6738 in combination with radiotherapy^40^. This approach may provide a pathway to effective combination with Top2 poisons with DNA damage repair inhibitors as well.

In conclusion, addition of DNA-PK inhibitors to Top2 poisons introduces a synergistic effect with the greatest impact on non-proliferating cells, sensitizing these otherwise resistant cells to result in greater or more persistent DNA damage and significant decreases in tumor cell survival. However, sensitization of non-cancerous, normal tissues and the overall greater dose-limiting toxicities observed with combination treatments suggest a more targeted therapeutic approach is necessary to maximise the potential benefit of combining Top2 poisoning with DNA-PK inhibition.

## Data Availability

All raw and processed data will be made available upon request to the corresponding author.
